# Supplementary material for: Flap Endonuclease 1 Endonucleolytically Processes RNA to Resolve R-Loops through DNA Base Excision Repair
Source: Genes (Basel). 2022 Dec 29;14(1):98. doi: 10.3390/genes14010098 (PMC9859040; doi:10.3390/genes14010098)
Supplement: Supplementary file 1 [file genes-14-00098-s001.zip › genes-1991883-supplementary.pdf]

genes

MDPI

Supplementary Materials and Data

Supplementary Table S1 The sequences of oligonucleotide substrates

Oligonucleotides	nt	Sequence (5′-3′)
Downstream/ Damaged strand		
D1	38	5′-pFGAT GAC GTA AAA GGA AAG AGA CGG AAG AGG AAG AAT TC-3′
D2	75	5′- CTC TCG GGG CTC TGG ATT GGC CAC CCA GTC TGC CCC CFG ATG ACG TAA AAG GAA AGA GAC GGA AGA GGA AGA ATT C-3′
R1	39	5′-rCrGrU rArCrG rCrGrG rArArU rArCrU rUrCrG rA TA CGT AGA CTT ACT CAT TGC-3′
R2	36	5′-rCrCrA rCrCrC rArGrU rCrUrG rCrCrC rCrCrG rGrArU rGrArC rGrUrA rArArA rGrGrA rArArG-3′
Template strand		
T1	60	5′-GGC AAT GAG TAA GTC TAC GTA TCG AAG TAT TCC GCG TAC GTA CGG ATG CTA GAT GAC TCG-3′
T2	77	5′-GGAA TTC TTC CTC TTC CGT CTC TTT CCT TTT ACG TCA TCC GGG GGC AGA CTG GGT GGC CAA TCC AGA GCC CCG AGA G -3′
Upstream strand		
U1	39	5′-CGA GTC ATC TAG CAT CCG TAC GTA CGC GGA ATA CTT CGA-3′
U2	29	5′-CGA GTC ATC TAG CAT CCG TAC GTA CGC GG-3′
U3	20	5′-CGA GTC ATC TAG CAT CCG TA-3′
U4	37	5′-CTC TCG GGG CTC TGG ATT GGC CAC CCA GTC TGC CCC C-3′

F:Tetrahydrofuran, THF
p: Phosphate
r: Ribonucleotide

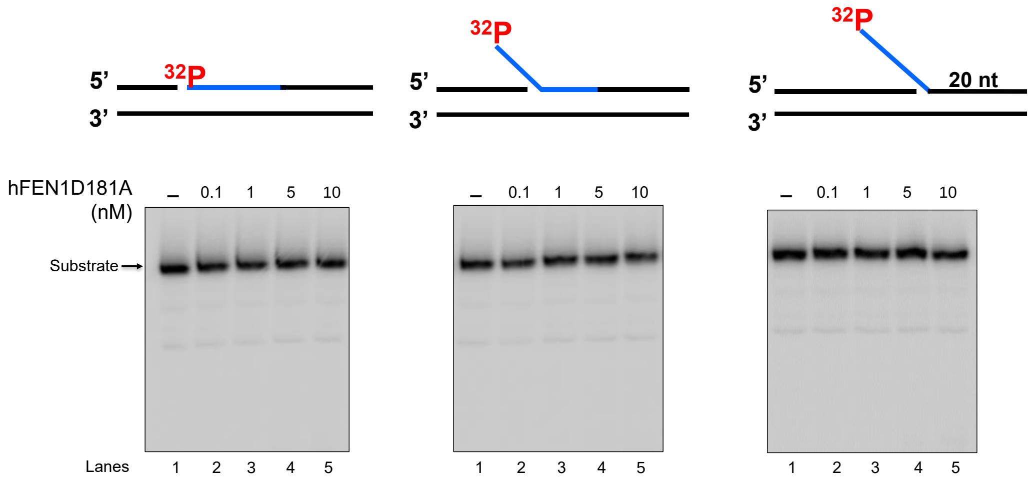

Supplementary Figure S1. FEN1 catalytic deficient mutant protein cannot cleave RNA. The RNA cleavage activity of the FEN1 catalytic deficient mutant protein, FEN1D181A was measured by incubating the mutant FEN1 protein at varying concentrations (0.1 nM-25 nM) with the substrates (25 nM) at 37°C for 30 min. Substrates were radioactively labeled at the 5′-end of the downstream strand containing an RNA (blue) and DNA (black) fragment on the same strand. Lane 1 represents substrate only. Lanes 2-5 represent the reaction mixture with increasing concentrations of FEN1D181A. Substrates and products were separated in a 15% urea-denaturing polyacrylamide gel and detected by phosphorimagery. The experiments were performed in triplicate.

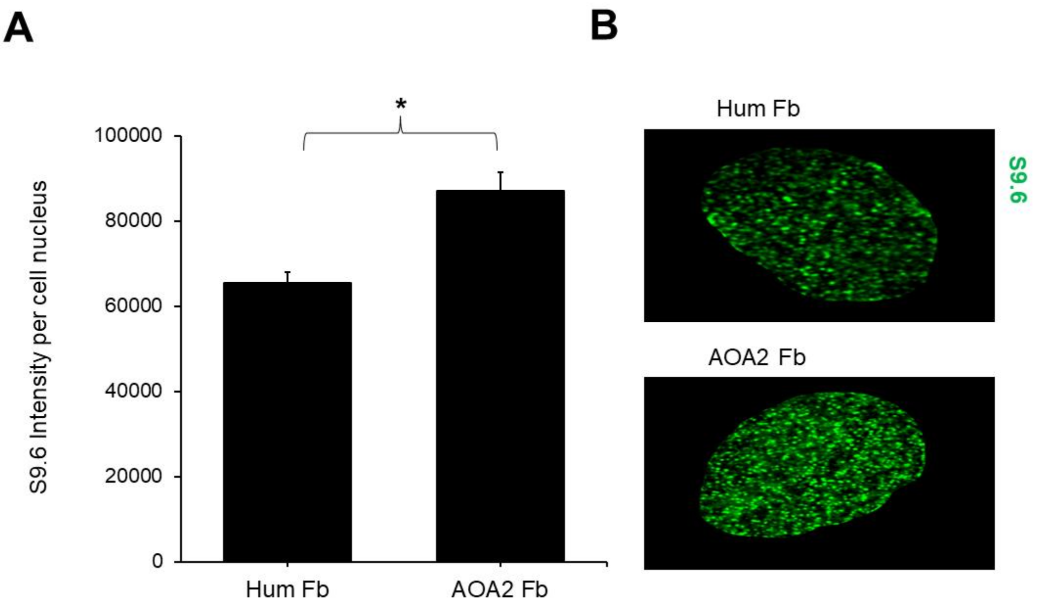

Supplementary Figure S2. RNA-DNA hybrids are more abundant in AOA2 cells than normal human fibroblasts. (A) AOA2 fibroblasts with senataxin deficiency exhibited more RNA-loops demonstrated by higher S9.6 antibody signal intensity than normal human fibroblasts (Hum Fb). The error bars are standard error of the mean. "*" indicates the statistical significance ($P < 0.05$ from Student t-test, 2 tailed, homoscedastic). (B) Immunofluorescent images of S9.6 antibody signal (green) in normal and AOA2 fibroblasts. Scale bar = 0.2 μm.

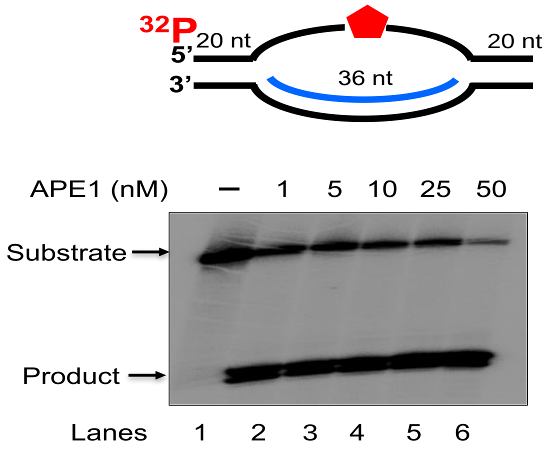

Supplementary Figure S3: APE1 can incise an abasic site in an R-loop. APE1 5′-incision of an abasic site (THF) on the non-template DNA strand of an R-loop was measured by incubating APE1 at varying concentrations (1 nM-50 nM) with 25 nM R-loop substrate at 37°C for 30 min. Lane 1 represents the substrate only. Lanes 2-6 represent the reaction mixture with various concentrations of APE1. The experiments were performed in triplicate.

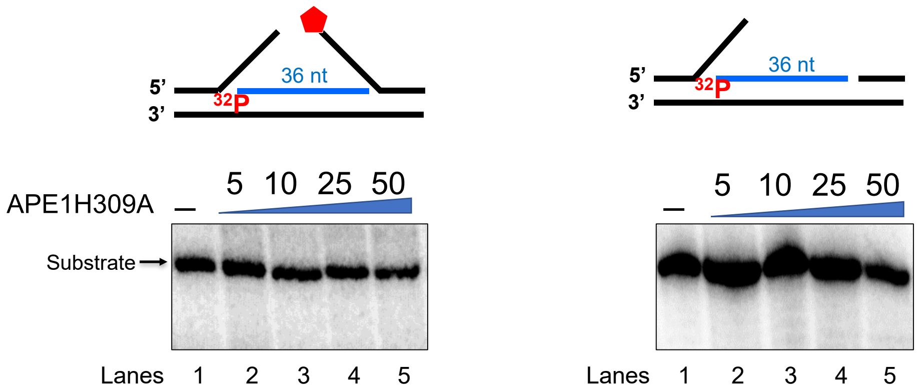

Supplementary Figure S4: APE1 catalytic mutant protein does not exhibit RNA cleavage activity on R-loop intermediates. The RNA cleavage activity of APE1H309A catalytic mutant protein on the R-loop intermediates generated during BER was examined by incubating various concentrations of the mutant protein from 5 nM to 50 nM with 25 nM substrate with a double DNA flap and the RNA:DNA hybrid or 25 nM substrate with a 3′-DNA flap along with the RNA:DNA hybrid. The enzyme and substrates were incubated at 37°C for 30 min. Lane 1 represents the substrate only. Lanes 2-5 represent the reaction mixture with various concentrations of APE1H309A with 25 nM substrates. The experiments were performed in triplicate.

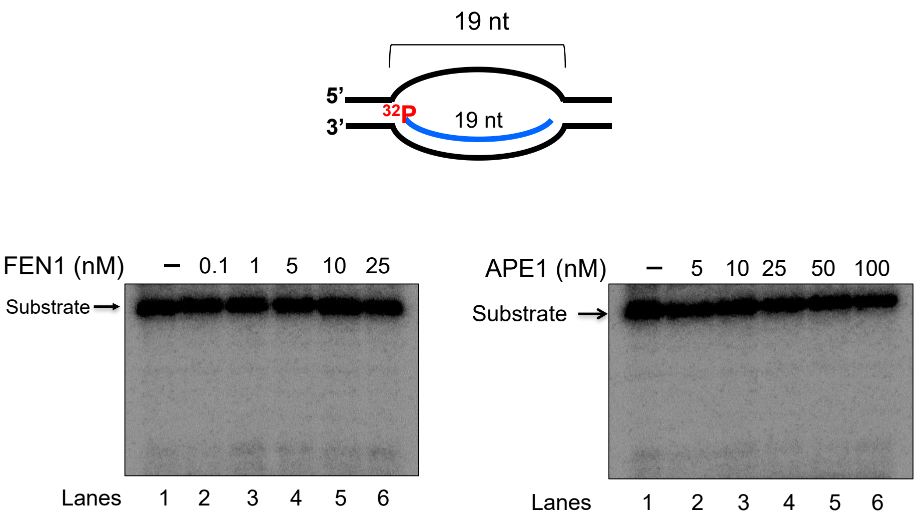

Supplementary Figure S5: FEN1 cannot make RNA cleavage on an R-loop in the absence of a DNA base lesion. To determine if FEN1 can cleave RNA in an R-loop without a DNA base lesion, various concentrations of FEN1 were incubated with 25 nM R-loop substrate with absence of a DNA base lesion at 37°C for 30 min. Lane 1 represents the substrate only. Lanes 2-5 represent the reaction mixture with various concentrations of FEN1 with the substrates. The experiments were performed in triplicate.
